# Supplementary material for: Spin-filtered measurements of Andreev bound states in semiconductor-superconductor nanowire devices
Source: Nat Commun. 2023 Oct 28;14:6880. doi: 10.1038/s41467-023-42026-7 (PMC10613242; doi:10.1038/s41467-023-42026-7)
Supplement: Supplementary file 1 — Supplementary Information [file 41467_2023_42026_MOESM1_ESM.pdf]

Supplementary information: Spin-filtered measurements of Andreev bound states in semiconductor-superconductor nanowire devices

David van Driel*,[1] Guanzhong Wang*,[1] Alberto Bordin,[1] Nick van Loo,[1] Francesco Zatelli,[1] Grzegorz P. Mazur,[1] Di Xu,[1] Sasa Gazibegovic,[2] Ghada Badawy,[2] Erik P. A. M. Bakkers,[2] Leo P. Kouwenhoven,[1] and Tom Dvir[1,*]

[1]*QuTech and Kavli Institute of NanoScience, Delft University of Technology, 2600 GA Delft, The Netherlands*

[2]*Department of Applied Physics, Eindhoven University of Technology, 5600 MB Eindhoven, The Netherlands*

(Dated: September 15, 2023)

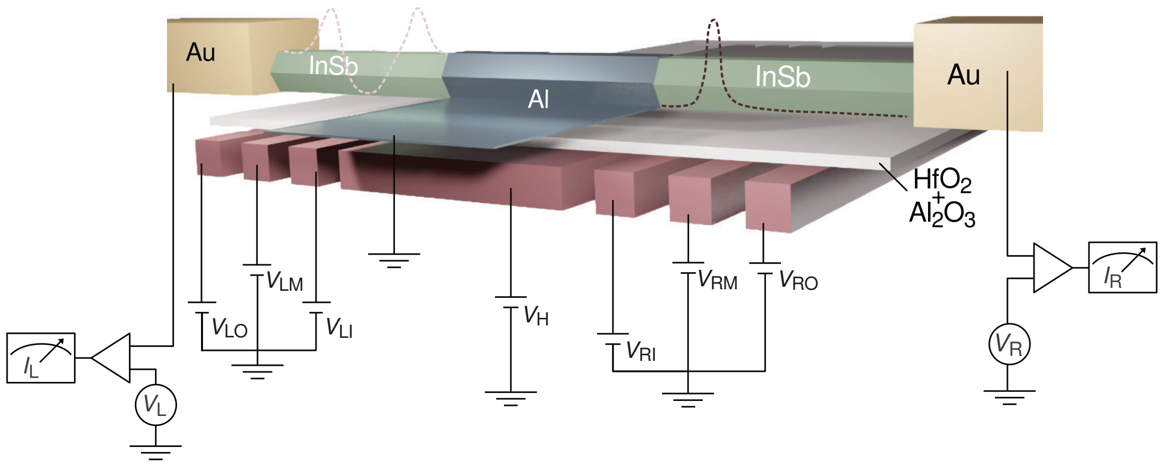

Supplementary Fig. 1. **Device schematic and measurement setup.** A quantum dot (QD) is defined by the three leftmost finger gates, V_{LO}, V_{LM} and V_{LI}, below the nanowire. The middle gate V_{H} controls the electrochemical potential of the hybrid InSb-Al nanowire. A tunnel junction is defined using V_{RI}. The voltages applied on each group of finger gates are schematically represented by the height of the voltage sources in the circuit diagram.

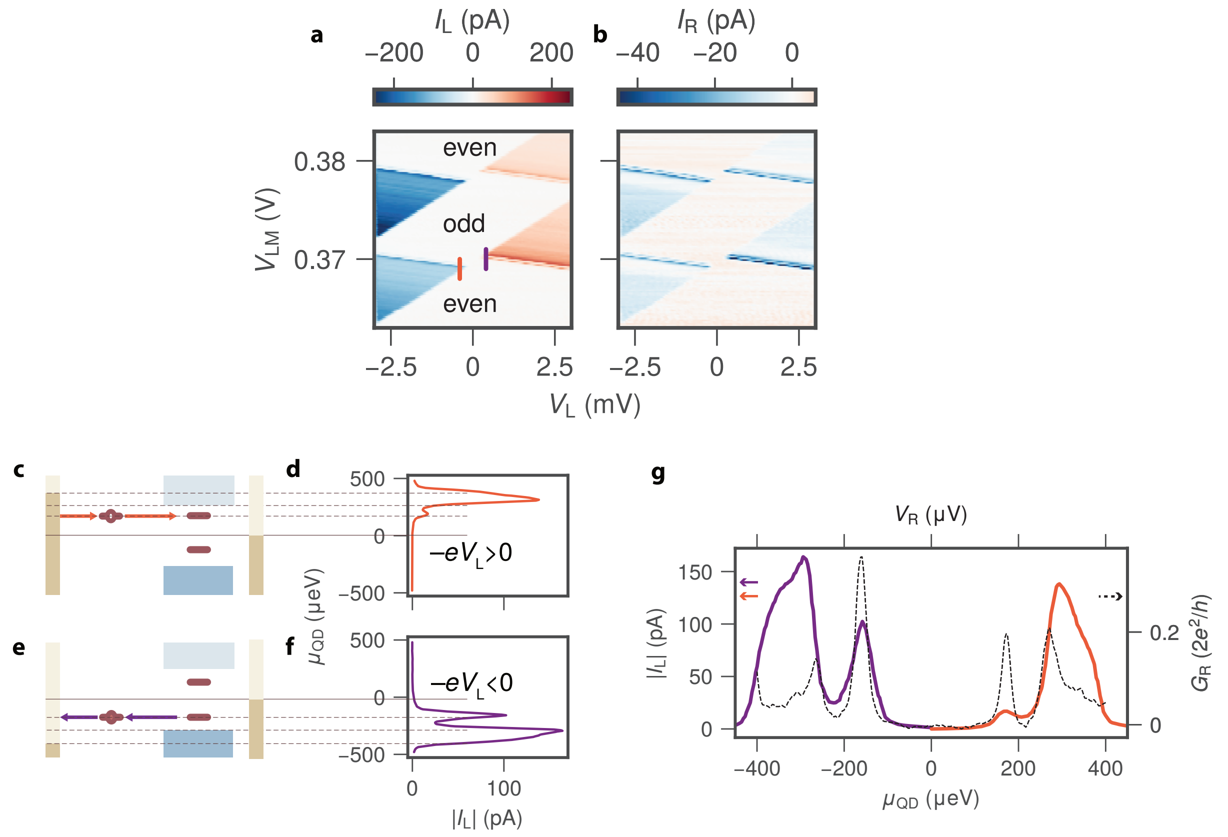

Supplementary Fig. 2. **Quantum dot spectroscopy. a., b.** I_{L} and I_{R} vs V_{L} and V_{LM} with $V_{\mathrm{R}} = 0$ showing the Coulomb diamond structure of the quantum dot (QD). **c., e.** Energy diagrams schematically depicting QD spectroscopy. Negative (panel c) or positive (panel e) V_{L} is set to fix the left lead potential above the bulk gap ($e|V_{\mathrm{L}}| > \Delta_{Al}$). μ_{QD} is tuned by varying V_{LM}. Current flows through the system when μ_{QD} aligns with the ABS or when aligned with the continuum states in the hybrid segment. **d. f.** Line-cut of $|I_L|$ taken from panel **a** at $V_{\mathrm{L}} = \pm 400\,\mu\mathrm{eV}$. The gate voltage V_{LM} has been converted to the electrochemical potential of the QD μ_{QD}. **g.** The positive energy section of panel **d.** (orange line), the negative energy section of panel **f.** (purple line) merged to show the full density of states measured by QD spectroscopy. In addition we show a linecut of G_{R} vs V_{R} (black dashed line), which was obtained with the tunnel junction when the QD was off-resonance and its lead grounded. See 4 for measurements of both polarities $V_{\mathrm{L}} = \pm 400\,\mu\mathrm{eV}$ presented separately.

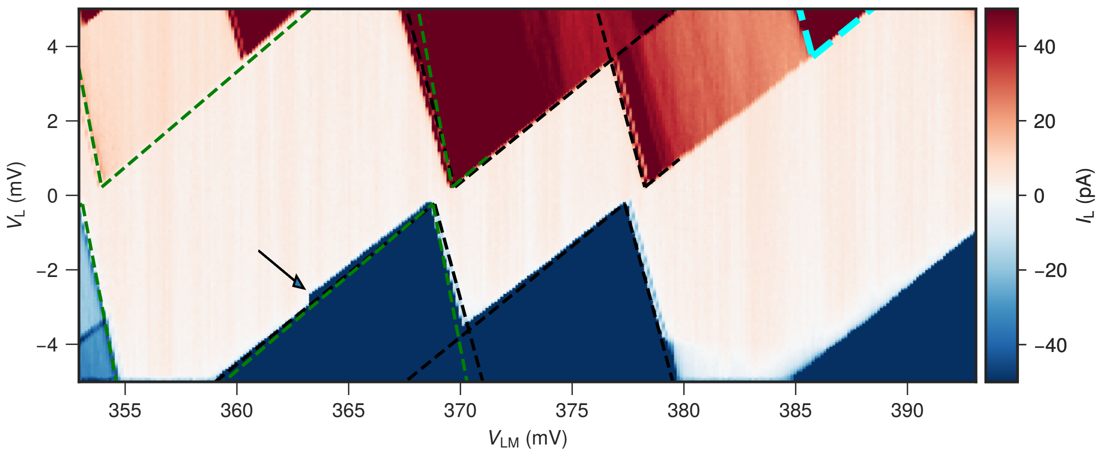

Supplementary Fig. 3. **Local Coulomb diamonds** A measurement of I_L for the quantum dot (QD) on the left side of the hybrid for $B = 0$. The bias V_L and dot gate V_LM are varied. There is a conversion factor between the QD electrochemical potential and the gate voltage. This is given by the capacitance between gate and dot relative to the total capacitance: $\alpha = C_G/C_{tot}$. Because Coulomb blockade blocks transport in a specific bias-gate window, it is possible to find this conversion factor. The black, dashed lines are a constant interaction model overlay for the $N+1$ Coulomb diamond, whose transitions are used as a spin filter throughout the paper [1]. From the constant interaction model we obtain $\alpha = 0.4 \pm .01$ and a charging energy $E_C = 3.4\,\mathrm{meV}$. The cyan lines indicate the next orbital level, for which we find an orbital level spacing of $\delta = 3.5\,\mathrm{meV}$. The green, dashed lines are a constant interaction model overlay for the Nth Coulomb diamond. From this model, we estimate an addition energy $E_\mathrm{add} = E_C + \delta \approx 6.9\,\mathrm{meV}$. This is in agreement with the previously mentioned level spacing and charging energy. We do note that the slopes of the green and black dashed lines do not completely agree. We attribute this to a gate jump that occurred at the voltage indicated by the arrow.

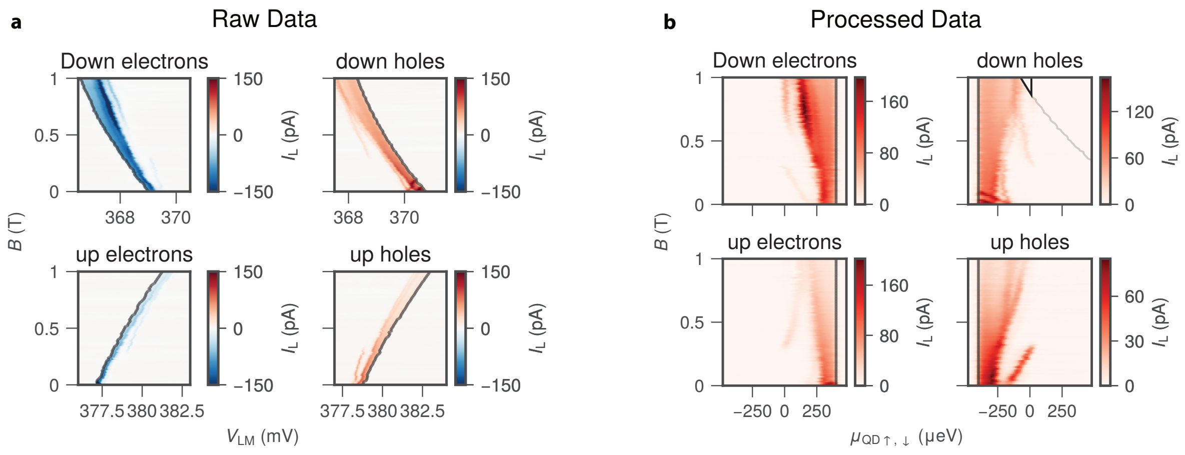

Supplementary Fig. 4. **Quantum dot (QD) spectroscopy data processing. a.** The raw data corresponding to main text Fig. 2. All electron data was measured at $-400\,\mu\text{eV}$, the hole data at $400\,\mu\text{eV}$. Magnetic field changes both the Andreev bound state (ABS) and QD level energies, hence, a slope between V_D and B is observed. I_L drops rapidly after the gate aligns the QD level with the bias window edge. This gate value V^0_LM is used as a reference to convert the gate voltage to the electrochemical potential of the QD using $\mu_\text{QD} = -(\alpha e(V_\text{LM} - V^0_\text{LM}) + eV_\text{L})$. The values found for V^0_LM are indicated by the black line in the plots. Note that the QD resonance moves out of the measurement range for high fields for spin-down holes. This results in missing data after converting V^0_LM to μ_QD. **b.** The processed data after the gate voltage is converted to the QD electrochemical potential. The field-dependence of the QD level energy is captured by V^0_LM, which allows us to arrive at a spectrum of the ABS. The black lines indicate $\mu_\text{QD} = -eV_\text{L}$. Everything above the gray line in the spin-down hole data is out of range for V_LM. For the most part, this is no problem, considering there is no hole transport for $\mu_\text{QD} > 0$. However, the gray line crosses zero for high fields, which leads to the missing data as indicated by the black triangle. This corresponds to the white triangle in main text Fig. 2.

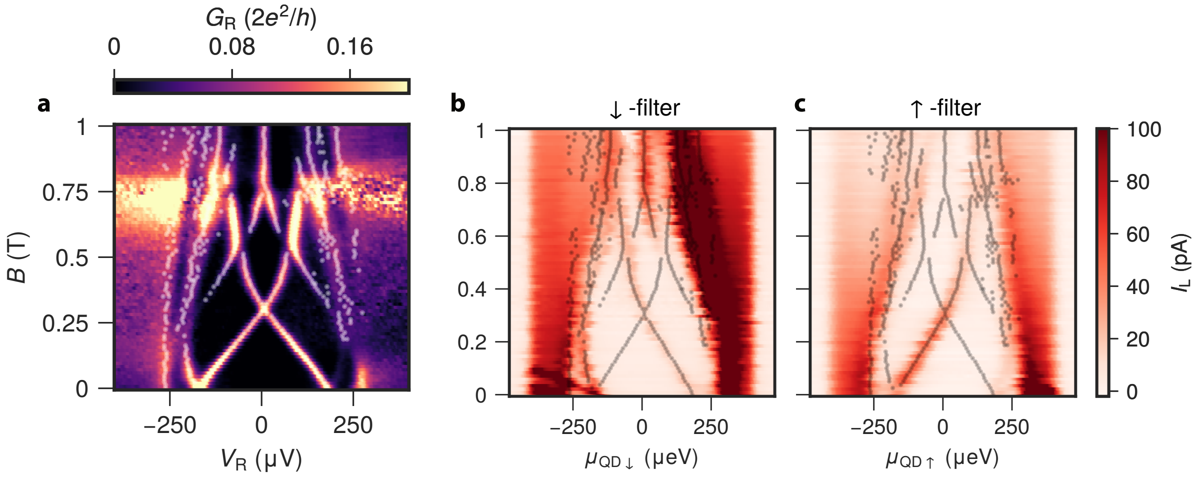

Supplementary Fig. 5. **Correlated quantum dot and tunneling spectroscopy (main text Fig. 2). a.** Tunneling spectroscopy of the hybrid for varying external field strength. Positions of peaks in tunneling spectroscopy are found using a standard peak-finding procedure provided by the SciPy python package [2]. **b., c.** I_L vs μ_{QD} and B using the ↓-filter (panel b) and ↑-filter (panel c). Both are overlaid with the peak energies found from tunneling spectroscopy in panel a.

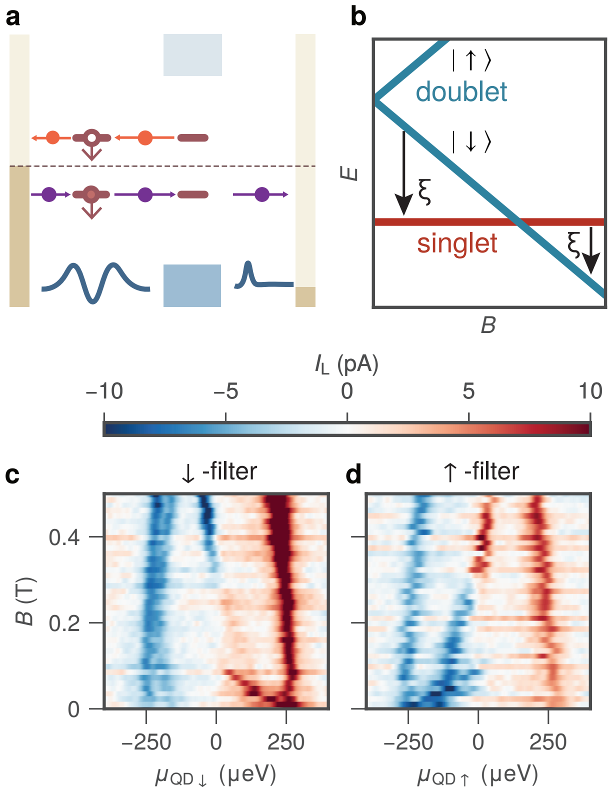

Supplementary Fig. 6. **Spin-polarization of the Andreev bound state relaxation. a.** Schematic of the transport cycle when the quantum dot (QD) lead is grounded and the tunnel junction bias is fixed at $eV_R = -400\,\mu V$. The non-polarized tunnel junction can both excite and relax the Andreev bound state (ABS). When the QD is on-resonance with the ABS, it provides a second path for the excited ABS to relax. **b.** Schematic energy diagram showing the evolution of the many-body spectrum with the applied magnetic field. The arrows illustrate the transitions from the first excited state to the ground state. **c., d** I_L for varying B and μ_{QD} using the QD as a $\downarrow$-filter (panel c) and $\uparrow$-filter (panel d). Similar to the results shown in Fig. 2, the spin-polarized measurement of the ABS shows only a single sub-gap peak traveling to lower energy and crossing zero at $B \approx 300\,\mathrm{mT}$. At low fields, when the ground state of the ABS is singlet, and the excited state is $|\downarrow\rangle$, the ABS can relax by emitting a down-polarized electron or an up-polarized hole. At higher applied fields, when the ground state becomes doublet, the selection rules reverse. Contrary to our discussion so far, the absence of current when the ABS and QD spin are opposite is not associated with a transport blockade, as the tunneling between the normal lead and the ABS proceeds regardless of the presence of the QD.

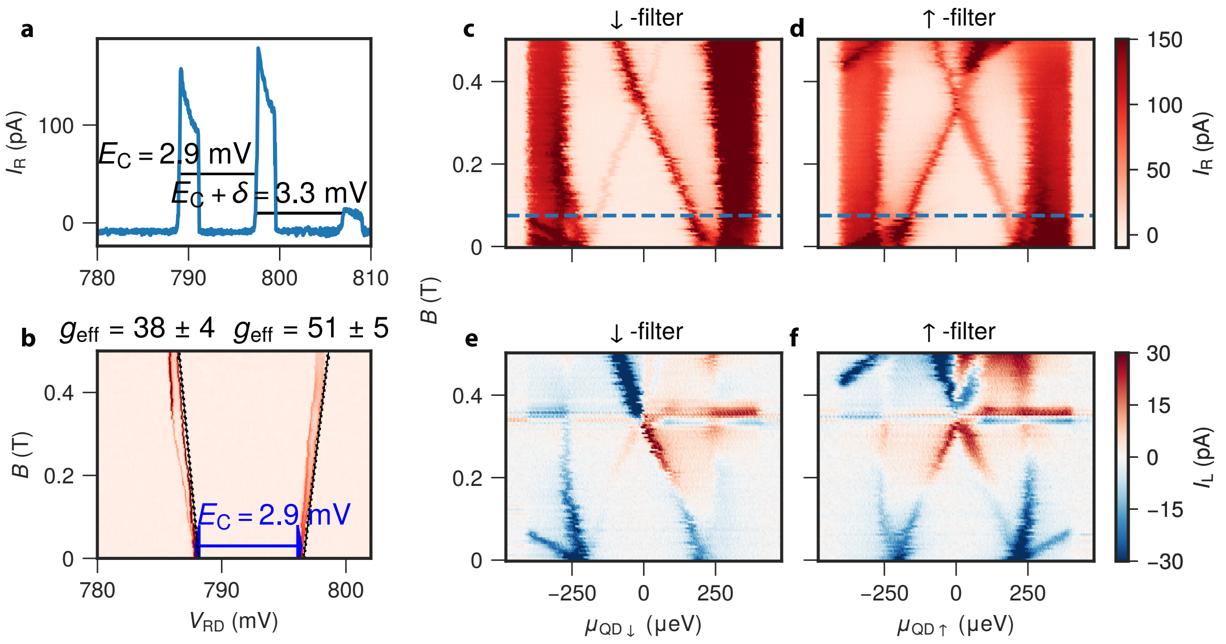

Supplementary Fig. 7. **Spin-polarized spectroscopy of a second device.** Measurements conducted on the device reported on in Ref. [3], which has an Al length of 180 nm, with an additional 2 Å of Pt grown at 30°. Here, the quantum dot (QD) formed on the right side of the Andreev bound state (ABS) served as the spin-filter for the spin-polarized spectroscopy. Compared to Ref. [3], here the barrier between the spin-polarized QD and the ABS was tuned to be much higher, reducing the transport between the QD and the ABS, and reducing the amount of spin-flip tunneling. **a.** The current through the QD I_{R} as a function of V_{RD}, for fixed $V_{\mathrm{R}} = 1\,\mathrm{mV}$. The two resonances observed around $V_{\mathrm{RD}} = 790\,\mathrm{mV}$ and $V_{\mathrm{RD}} = 798\,\mathrm{mV}$ serve as our ↓ and ↑ spin filters respectively. Note the additional resonance at $V_{\mathrm{RD}} = 808\,\mathrm{mV}$, that we attribute to the next orbital level. Spin-orbit coupling on the QD can give rise to spin mixing decreasing the efficiency of the ↑ spin filter. **b.** The current through the QD I_{R} as a function of V_{RD} and B, for fixed $V_{\mathrm{R}} = 0.3\,\mathrm{mV}$. The two resonances evolve with the expected trajectory showing their spin polarization. We determine E_{C} and g_{eff} using a lever arm of 0.34 for the ↓-filter and 0.37 for the ↑-filter. These are also used for panels c-f. **c., d.** I_{R} vs μ_{QD} and B using the QD as a ↓-filter (panel a) and ↑-filter (panel b). The QD acts as a spin filter for $B > 75\,\mathrm{mT}$, indicated by the blue line. Below the blue line, both QD spins are within the bias window and participate in transport. We note that the ↓ filter shows the expected behavior as discussed in the main text, with some leakage current resulting from spin-flip tunneling or spin-orbit effect in the ABS itself. The ↑ filter shows little signs of polarization. We attribute this to strong spin mixing within the QD making it a poor spin-filter. **e., f.** I_{L} vs μ_{QD} and B using the QD as a ↓-filter (panel c) and ↑-filter (panel d).

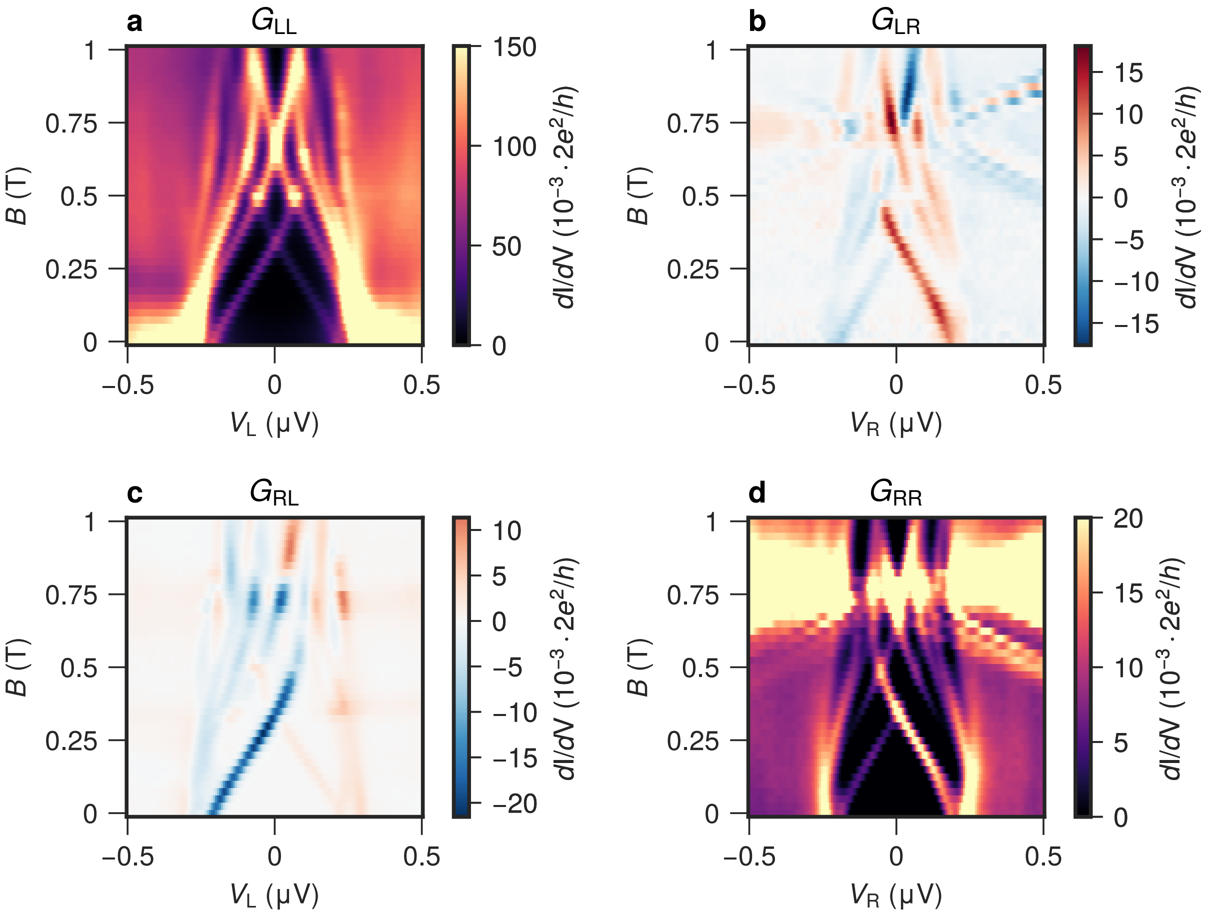

Supplementary Fig. 8. **Full conductance matrix of the Andreev bound state studied in the main text for different gate settings.** See ref [4] for details on the measurement technique. The gates forming a quantum dot to the left of the hybrid are reconfigured as a tunnel junction to measure non-local conductance. **a.** Local tunneling spectroscopy on the left of the hybrid $G_{\mathrm{LL}} = dI_{\mathrm{L}}/dV_{\mathrm{L}}$ for varying B. **b.** Non-local conductance $G_{\mathrm{LR}} = dI_{\mathrm{L}}/dV_{\mathrm{R}}$ for varying B. **c.** Non-local conductance $G_{\mathrm{RL}} = dI_{\mathrm{R}}/dV_{\mathrm{L}}$ for varying B. **d.** Local tunneling spectroscopy on the right of the hybrid $G_{\mathrm{RR}} = dI_{\mathrm{R}}/dV_{\mathrm{R}}$ for varying B.

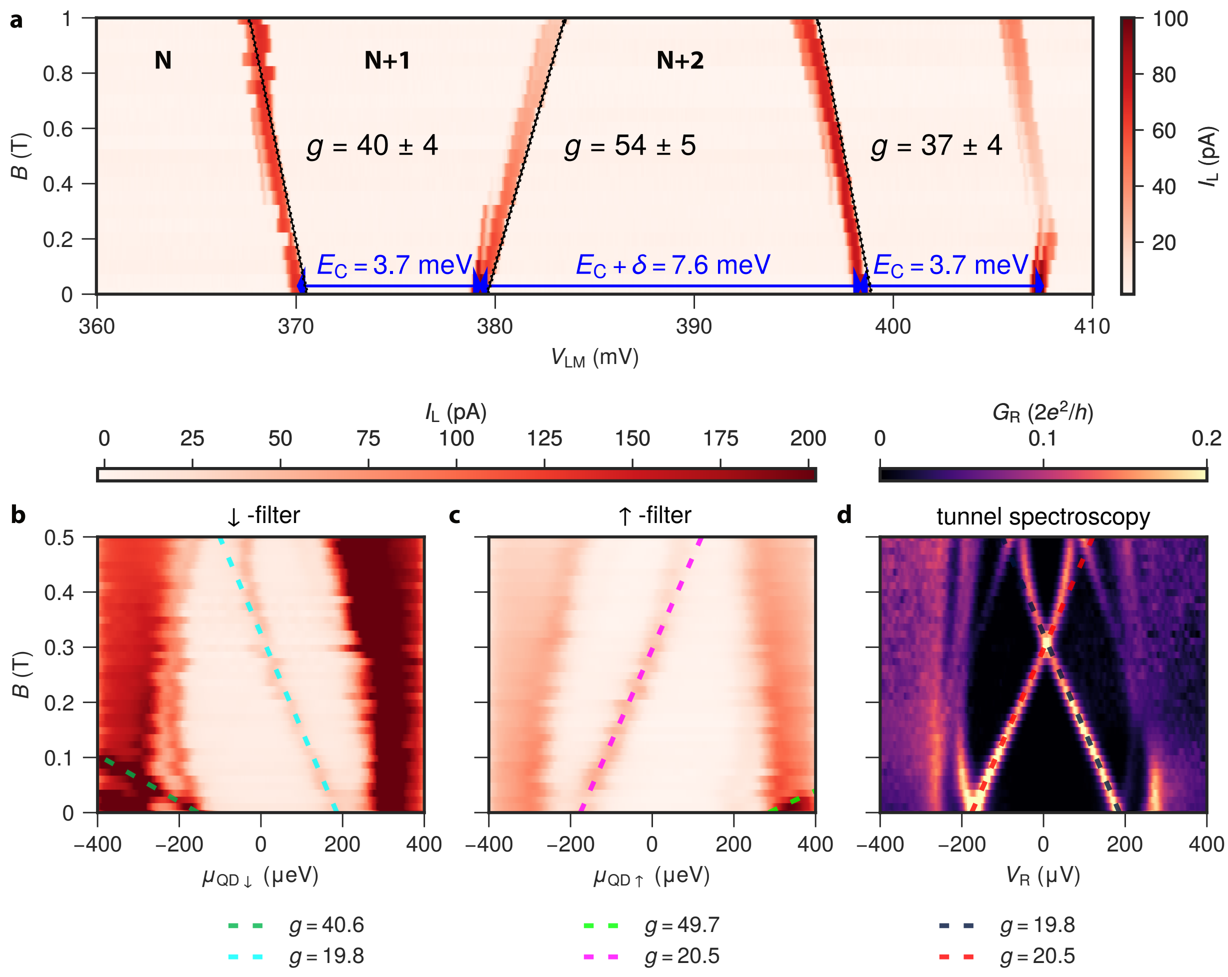

Supplementary Fig. 9. g **factors and quantum dot level spacing. a.** A measurement of I_L for the quantum dot (QD) on the left side of the hybrid for varying B and dot gate $V_{\rm LM}$. Horizontal lines estimate spacing between adjacent QD levels. A lever arm of 0.4 found from Fig. 3 is used to convert gate values into units of energy, and calculate addition energies. The sloped lines estimate the g factor of the QD levels. **b., c.** A zoom in of Fig. 2b, c with dashed lines estimating the g factor of the QD and lowest-lying Andreev bound state (ABS). **d.** The lines corresponding to the ABS g factor overlaid on top of the tunnel spectroscopy of Fig. 2a.

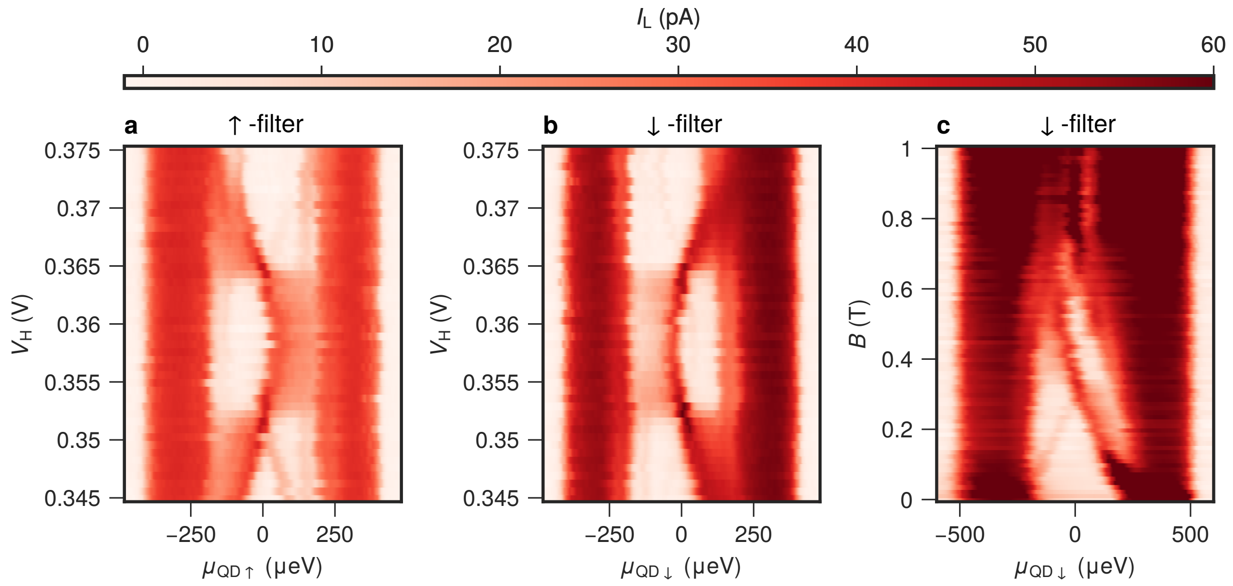

Supplementary Fig. 10. **Spin-polarized quantum dot spectroscopy of an Andreev bound state for different gate settings.** All main text figures were obtained for $V_{\mathrm{LI}} = 135\,\mathrm{mV}$, while here we set $V_{\mathrm{LI}} = 168\,\mathrm{mV}$.

a., b. I_{L} vs μ_{QD} and V_{H} using the quantum dot (QD) as a ↓-filter (panel a) and ↑- filter (panel b) for $B = 400\,\mathrm{mT}$. Peaks in I_{L} can be seen for both negative and positive μ_{QD}, indicating incomplete spin-polarization. We further see signatures of inelastic tunneling compatible with the higher tunneling rate between the QD and the Andreev bound state [5]. **c.** I_{L} vs μ_{QD} and B at using the QD as a ↓-filter for $V_{\mathrm{H}} = 356\,\mathrm{mV}$. We observe incomplete spin-polarization as in panels a and b.

* tom.dvir@gmail.com

[1] Kouwenhoven, L. P., Austing, D. & Tarucha, S. Few-electron quantum dots. *Reports on Progress in Physics* **64**, 701 (2001).

[2] Virtanen, P. *et al.* Scipy 1.0: fundamental algorithms for scientific computing in python. *Nature Methods* **17**, 261–272 (2020).

[3] Wang, G. *et al.* Singlet and triplet cooper pair splitting in hybrid superconducting nanowires. *Nature* 1–6 (2022).

[4] Ménard, G. C. *et al.* Conductance-Matrix Symmetries of a Three-Terminal Hybrid Device. *Physical Review Letters* **124**, 036802 (2020). 1905.05505.

[5] Fujisawa, T. *et al.* Spontaneous emission spectrum in double quantum dot devices. *Science* **282**, 932–935 (1998).
